# Prenatal Vitamin D Intake, Cord Blood 25-Hydroxyvitamin D, and Offspring Body Composition: The Healthy Start Study

**DOI:** 10.3390/nu9070790

**Published:** 2017-07-22

**Authors:** Katherine A. Sauder, Hallie J. Koeppen, Allison L.B. Shapiro, Kathryn E. Kalata, Alexandra V. Stamatoiu, Brandy M. Ringham, Deborah H. Glueck, Jill M. Norris, Dana Dabelea

**Affiliations:** 1 Department of Pediatrics, University of Colorado School of Medicine, Aurora, CO 80045, USA; Kathryn.kalata@ucdenver.edu (K.E.K.); Dana.Dabelea@ucdenver.edu (D.D.); 2 Department of Epidemiology, Colorado School of Public Health, Aurora, CO 80045, USA; Hallie.koeppen@mt.gov (H.J.K.); Allison.shapiro@ucdenver.edu (A.L.B.S.); Alexandra.stamatoiu@ucdenver.edu (A.V.S.); Jill.Norris@ucdenver.edu (J.M.N.); 3 Department of Biostatistics and Informatics, Colorado School of Public Health, Aurora, CO 80045, USA; Brandy.ringham@ucdenver.edu (B.M.R.); Deborah.glueck@ucdenver.edu (D.H.G.)

**Keywords:** vitamin D, pregnancy, birth, body composition, adiposity

## Abstract

Vitamin D deficiency in pregnancy may be associated with increased offspring adiposity, but evidence from human studies is inconclusive. We examined associations between prenatal vitamin D intake, 25-hydroxyvitamin D (25(OH)D) in cord blood, and offspring size and body composition at birth and 5 months. Participants included 605 mother-offspring dyads from the Healthy Start study, an ongoing, pre-birth prospective cohort study in Denver, Colorado, USA. Prenatal vitamin D intake was assessed with diet recalls and questionnaires, and offspring body composition was measured via air displacement plethysmography at birth and 5 months. General linear univariate models were used for analysis, adjusting for maternal age, race/ethnicity, pre-pregnancy body mass index (BMI), offspring sex, and gestational age at birth. Non-Hispanic white race, lower pre-pregnancy BMI, higher prenatal vitamin D intake, and summer births were associated with higher cord blood 25(OH)D. Higher 25(OH)D was associated with lower birthweight (*β* = –6.22, *p* = 0.02), but as maternal BMI increased, this association became increasingly positive in direction and magnitude (*β* = 1.05, *p* = 0.04). Higher 25(OH)D was also associated with lower neonatal adiposity (β = –0.02, p < 0.05) but not after adjustment for maternal BMI (*β* = –0.01, *p* = 0.25). Cord blood 25(OH)D was not associated with offspring size or body composition at 5 months. Our data confirm the hypothesis that vitamin D exposure in early life is associated with neonatal body size and composition. Future research is needed to understand the implications of these associations as infants grow.

## 1. Introduction

The fetal programming hypothesis posits that prenatal malnutrition, including micronutrient deficiencies, increases offspring risk for obesity throughout the lifespan [1]. Approximately 33% of pregnant women in the United States have vitamin D deficiency, defined as 25-hydroxyvitamin D (25OHD) < 50 nmol/L, even though 73% report using vitamin D-containing prenatal supplements [2]. Maternal 25(OH)D freely crosses the placenta and is the only source of vitamin D available to the developing fetus [3]. Prenatal vitamin D deficiency has been associated with pre-term birth [4,5], low birthweight [5,6], and small for gestational age offspring [5,6,7], while prenatal vitamin D supplementation has been shown to increase offspring birth weight and length [8]. Prenatal vitamin D exposure may also influence offspring adiposity through reduced availability of the biologically active vitamin D metabolite, 1α, 25-dihydroxyvitamin D, which inhibits parathyroid hormone and intracellular calcium, thereby affecting lipogenesis and lipolysis [9,10,11,12,13].

Current human evidence supporting the association between prenatal vitamin D intake, neonatal vitamin D status, and offspring adiposity is mixed. While numerous studies have reported that higher maternal vitamin D intake (from food and/or dietary supplements) is associated with increased prenatal and/or cord blood 25(OH)D levels [14,15,16,17], it is not clear whether this translates to an effect on neonatal adiposity. Of the existing studies that have measured both 25OHD and neonatal adiposity, some report that lower 25(OH)D is associated with increased adiposity in neonates, [18,19], others that higher 25(OH)D is associated with increased neonatal adiposity [20,21,22], and still others that there is no association at all [23,24]. Evidence for an association with adiposity at 6–10 years of age is equally mixed [21,25,26,27]. There is also a notable lack of study on body composition during infancy, a time in which postnatal exposures (infant vitamin D supplementation, vitamin D-fortified formula use) may also play a pivotal role in adiposity development.

Prior studies vary strongly in methodology, particularly in regard to assessment of prenatal vitamin D intake, timing and source of 25(OH)D measurements, a method for assessing neonatal adiposity, and consideration of other contributing factors such as maternal obesity. The heterogeneity of methods makes it difficult to draw conclusions from the currently available evidence. In addition, few studies examined whether gestational weight gain or body mass index (BMI) modify the associations between prenatal vitamin D intake, neonatal vitamin D status, and offspring adiposity. Given that levels of 25(OH)D decrease with increasing body mass index [28,29,30,31], even among pregnant women [20,32,33,34], it is plausible that an association between 25(OH)D and neonatal outcomes may be attenuated among women with obesity due to decreased bioavailabilty of 25(OH)D [28].

Thus, the purpose of this analysis was to examine the associations between prenatal vitamin D intake, neonatal vitamin D status, and early life body composition. We also investigated whether the associations were modified by maternal pre-pregnancy BMI and gestational weight gain (GWG).

## 2. Methods

### 2.1. Participants

The Healthy Start Study is an ongoing observational pre-birth cohort of mother-offspring dyads based in Denver, Colorado. Pregnant women were recruited from the Obstetric clinics at the University of Colorado Anschutz Medical Campus from 2009 to 2014. During this time, all women who presented at the clinics were routinely invited to participate in research studies. Contact information of women who indicated interest was provided to study staff, who contacted them to confirm eligibility. Women also responded directly to study advertisements that were posted around the Medical Campus. Women were eligible for the study if they were pregnant with a single fetus, at least 16 years old, had no serious chronic medical conditions (diabetes, cancer, etc.), and no history of adverse obstetric outcomes (prior stillbirth). Enrolled participants completed research visits in early pregnancy (median 17 weeks), mid-pregnancy (median 27 weeks), at delivery (median 1 day), and postnatally (median 5.4 months). The study protocol was approved by the Colorado Multiple Institutional Review Board, and all participants provided written informed consent.

### 2.2. Assessment of Maternal Vitamin D Intake

Prospective data on vitamin D intake from food and dietary supplements was collected from early pregnancy through 5 months postnatally. Prenatal dietary intake was assessed using the Automated Self-Administered 24-hour Dietary Recall (ASA24) system created by the National Cancer Institute (NCI) [35]. The food list is derived from the current United States Department of Agriculture Food and Nutrient Database for Dietary Studies, and the output includes individual food and daily estimates of energy, macronutrients, micronutrients, and MyPyramid Food Equivalents. Women completed recalls at the early and mid-pregnancy research visits, and were asked to complete up to six additional recalls during pregnancy. Monthly calls were made by the University of North Carolina at Chapel Hill to remind participants to complete their dietary recalls at home. On average, participants completed two recalls over the pregnancy period (range: 1–8), with 76% having at least two diet recalls. Women also completed a Food Propensity Questionnaire (FPQ) at the mid-pregnancy and delivery visits, which measured frequency of consumption of major food groups (grains, fish, red meat, dairy, fruits, etc.) in the prior 3 months. The purpose of the FPQ was to capture intake of periodically consumed foods that could notably contribute to dietary intake but may not have been consumed in the 24-hour period assessed by the ASA24. We used the NCI’s measurement error model to derive usual dietary intake throughout pregnancy from the ASA24 recalls and FPQ [36,37,38]. The NCI method uses a two-part non-linear mixed effects model to estimate nutrient intake from a combination of single and multiple dietary recalls. An a priori list of covariates derived from pregnancy and nutrition literature was included in the NCI model (prenatal smoking, pre-pregnancy obesity, gravidity). Using this model, we estimated usual daily intake of vitamin D from food sources during pregnancy (IU/day).

At each research visit, women self-reported dietary supplement use in the 12 weeks prior to conception (at the early pregnancy visit), since the prior visit (at the mid-pregnancy and delivery visits), and since delivery (at the 5-month postnatal visit). When applicable, women were asked about starting and stopping date, dosages, and brand/type. We calculated duration of use and average daily servings (i.e. dose) for each supplement, and used manufacturer data on nutrient composition to estimate average daily dose of vitamin D from dietary supplements (IU/day) during pregnancy. Daily vitamin D from dietary supplements was combined with daily vitamin D from food sources to estimate total oral intake of vitamin D in pregnancy.

### 2.3. Assessment of Offspring Vitamin D Intake

At the postnatal research visit, women provided information on breastfeeding and formula use. We quantified breastfeeding with breastmilk-months [39], a metric that reflects both duration and exclusivity of breastfeeding. For example, 6 months of exclusive breastfeeding equates to 6 breastmilk-months while 4 months of exclusive breastfeeding followed by 2 months of 50% breastmilk and 50% formula equates to 5 breastmilk-months. This variable was used as proxy for offspring vitamin D intake from food, given that breastmilk contains little-to-no vitamin D and all infant formulas in the United States are required to contain at least 400 IU/L [40].

Regarding infant supplements, mothers also reported use of infant fluoride, multivitamins, iron, and any other supplements since birth. Mothers were asked to specify “other supplements” (e.g., vitamin D, probiotics), and provide starting date, stopping date, and doses per week for all infant supplements. We did not collect data on brands, and thus were unable to match reported supplements with manufacturer’s nutrient composition data for any infant supplements reported. Instead, we quantified infant supplement use by calculating the weeks of daily use for each supplement, as has been done previously [41]. For example, an infant who received one dose per day of a multivitamin supplement for 20 weeks between birth and the postnatal visit would have 20 weeks of daily multivitamin supplement use. An infant who received one dose every other day for 20 weeks would have 10 weeks of daily multivitamin supplement use. We estimated weeks of daily vitamin D supplement use for offspring by summing the weeks of daily vitamin D use (when reported by the mother as an “other” supplement) and weeks of daily multivitamin use, as infant multivitamin supplements typically contain vitamin D [42].

### 2.4. Assessment of 25(OH)D

Umbilical cord blood samples were obtained at delivery and processed by the Colorado Clinical and Translational Science Institute Core Laboratory. Serum samples were stored at –80 °C for up to 6.4 years (mean 4.8 ± 0.6 years). Thawed serum samples were analyzed in one batch with the ImmunoDiagnostic Systems iSYS 25OHD assay using chemiluminescence technology. This assay has been certified in the Vitamin D Standardization Program (VDSP) [43,44], validated against liquid chromatography-tandem mass spectrometry methods [45,46], and has 100% cross-reactivity with 25(OH)D_2_ and 25(OH)D_3_ [47]. Separate estimates of 25(OH)D_2_ and 25(OH)D_3_ were not available.

### 2.5. Assessment of Offspring Size and Body Composition

Offspring size and composition was measured at delivery (median 1 day) and postnatally (median 5.1 months) with whole body air displacement plethysmography (PEA POD, COSMED, Rome, Italy). This device uses a two-compartment model to estimate fat mass (adipose tissue; g and percent of total mass) and fat-free mass (water, bone, etc.; g and percent of total mass). Plethysmography has excellent validity and reliability in infants [48,49,50]. Trained personnel conducted two measurements on each infant, with a third measurement taken if the percent fat mass differed by >2%. The average of the two closest readings was used for analysis of total mass (g), fat-free mass (g), fat mass (g), and adiposity (%).

### 2.6. Covariates

Potential covariates were identified through a review of relevant literature [20,21,26,32,51,52,53,54,55]. Season of birth was classified as winter (December–February), spring (March–May), summer (June–August), or fall (September–November), and used as a proxy in our models for endogenous production of 25(OH)D driven by exposure to sunlight. Maternal pre-pregnant weight was abstracted from the medical record (91%) or collected from self-reported data (9%) at study enrollment. Height was measured at enrollment and used to calculate body mass index (kg/m^2^). GWG (kg) was calculated from last recorded weight during pregnancy and pre-pregnant weight. Maternal age at delivery was calculated from the self-reported maternal date of birth and date of delivery of the offspring. Maternal race/ethnicity and household income were obtained from self-report at enrollment. Offspring gestational age at birth was estimated via prenatal ultrasound measurements and/or self-reported first day of last menstrual period. Offspring sex was obtained from maternal report at the delivery visit.

### 2.7. Statistical analyses

**Figure 1:**
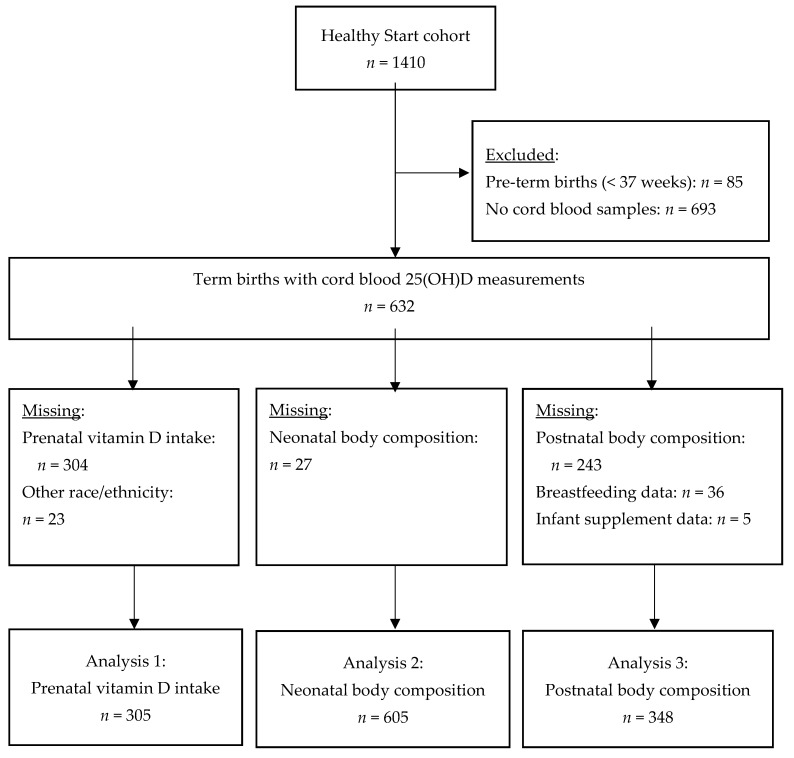
Participant flow diagram.

Three separate analyses were conducted for which the respective analytic samples were constructed to maximize sample size (Figure 1). Mother-offspring dyads were eligible for the analyses if offspring were born ≥37 weeks gestation, had 25(OH)D measured in cord blood, and had at least one of the following: complete data on maternal intake of vitamin D during pregnancy from food and dietary supplements, body composition measured at birth, or body composition measured postnatally. For the vitamin D intake analysis, we excluded offspring who identified as a race/ethnicity other than non-Hispanic white, non-Hispanic black, or Hispanic due to small cell sizes. For the postnatal analysis, we additionally excluded offspring who were missing breastfeeding/formula data or infant dietary supplement use data.

All analyses were conducted in SAS 9.4 (SAS Institute, Cary, NC, USA) using general linear univariate models in a multi-step approach that included examination of potential effect modifiers. When the interaction terms were statistically non-significant, they were removed from the model and the main effects interpreted. Statistical significance was set at 0.05. Examination of jackknifed studentized residuals confirmed the assumptions of normality and homoscedasticity. We report beta estimates (standard errors; SE) unless otherwise indicated.

The first analysis evaluated the association of maternal oral intake of vitamin D during pregnancy with cord blood 25(OH)D. This model was adjusted for season of birth, maternal age, pre-pregnancy BMI, race/ethnicity, household income, GWG, and gestational age at birth and considered the following interaction terms: vitamin D intake × BMI, vitamin D intake × GWG, vitamin D intake × race/ethnicity, season × BMI, season × GWG, and season × race/ethnicity.

The second analysis evaluated the association of cord blood 25(OH)D with total mass (g), fat-free mass (g), fat mass (g), and adiposity (% fat mass) at birth. We fit separate simple models for each neonatal outcome that were adjusted for maternal age, race/ethnicity, household income, gestational age at birth, and offspring sex. A simple + BMI model included maternal pre-pregnancy BMI and an interaction term of 25(OH)D × BMI. A simple + GWG model included maternal GWG and an interaction term of 25(OH)D × GWG.

The third analysis evaluated the association of cord blood 25(OH)D with total mass (g), fat-free mass (g), fat mass (g), and adiposity (% fat mass) at the 5-month postnatal visit. As above, we fit a separate model for each postnatal outcome that was adjusted for maternal age, race/ethnicity, household income, gestational age at birth, offspring sex, and offspring age at the postnatal visit. We examined the simple + BMI and simple + GWG models in the same manner as the neonatal analysis. We also examined a simple + breastfeeding model that included breastmilk months and an interaction term of 25(OH)D × breastmilk−months, and a simple + offspring supplements model that included weeks of daily infant supplement use and interaction term of 25(OH)D × supplement use.

## 3. Results

Complete data were available for 305 dyads for the vitamin D intake analysis, 605 dyads for the neonatal analysis, and 348 dyads for the postnatal analysis (Figure 1). Maternal–offspring characteristics are presented in Table 1. The analytic cohort was similar to the full Healthy Start cohort (*n* = 1410) in terms of maternal age, race/ethnicity, education, pre-pregnancy BMI, and gestational age at birth (data not shown).

### 3.1. Analysis 1: Vitamin D intake (Table 2)

All interaction terms were non-significant and removed from the model. Every 100 IU/day increase in oral vitamin D was associated with a 0.6 (0.2) nmol/L increase in cord blood 25(OH)D (*p* = 0.01). Every 5 kg/m^2^ increase in maternal body mass index (BMI) was associated with a 3.2 (0.8) nmol/L decrease in cord blood 25(OH)D (*p* < 0.001). Compared to offspring born in the summer months, offspring born in the fall months had 25(OH)D levels lower by 6.8 (2.9) nmol/L (*p* = 0.02), offspring born in the winter months had 25(OH)D levels lower by 12.1 (3.1) nmol/L (*p* < 0.001), and offspring born in the spring months had 25(OH)D levels lower by 10.4 (2.9) nmol/L (*p* < 0.001). Compared to non-Hispanic white offspring, non-Hispanic black offspring had 25(OH)D levels that were 15.6 (3.4) nmol/L lower at birth (*p* < 0.001), and Hispanic offspring had 25(OH)D levels that were 8.2 (3.0) nmol/L lower at birth (*p* = 0.006). Maternal age at delivery, household income, gestational weight gain (GWG), and gestational age at birth were not significantly associated with 25(OH)D (data not shown).

### 3.2. Analysis 2: Neonatal Body Size and Composition (Table 3)

In the simple model, increasing 25(OH)D was associated with decreased total mass at birth (β = −1.44, SE = 0.76), but this did not reach statistical significance (*p* = 0.06). In the simple + BMI model, the interaction of 25(OH)D with BMI was statistically significant for the outcome of total mass (β = 1.05, SE = 0.50, *p* = 0.04). As maternal BMI increased, the association between 25(OH)D and total mass at birth became increasingly positive in direction and magnitude: for women with a lower BMI, higher 25(OH)D was associated with a lower total mass, but for women with a higher BMI, higher 25(OH)D was associated with a higher total mass. Similarly, in the simple + GWG model, the interaction of 25(OH)D with GWG was statistically significant (β = 0.26, SE = 0.11, *p* = 0.02), with the association between 25(OH)D and total mass becoming increasingly positive in direction and magnitude as GWG increased. SE: standard error

There was no association of 25(OH)D with fat-free mass (*β* = –0.91, SE = 0.59, *p* = 0.12) in the simple model. The interaction of 25(OH)D with BMI did not reach significance (*β* = 0.68, SE = 0.39, *p* = 0.08) in the simple + BMI model, but the interaction with GWG was significant in the simple + GWG model (*β* = 0.20, SE = 0.09, *p* = 0.02). As above, the association between 25(OH)D and fat-free mass became increasingly positive in direction and magnitude as GWG increased.

Here, 25(OH)D was not associated with fat mass in the simple model (*β* = –0.48, *p* = 0.11), nor were the interactions of 25(OH)D with BMI or GWG significant in the respective models (*β* = 0.35, SE = 0.19, *p* = 0.07 and *β* = 0.06, SE = 0.04, *p* = 0.16). Increasing 25(OH)D was associated with decreasing adiposity in the simple model (*β* = -0.02, SE = 0.01, *p* = 0.046). The interactions of 25(OH)D with BMI and GWG were not significant (*β* = 0.01, SE = 0.01, *p* = 0.16 and *β* = 0.001, SE = 0.001, *p* = 0.32). After adjustment for BMI, the main effect of 25(OH)D with adiposity was no longer significant (*p* = 0.25). After adjustment for GWG, the main effect of 25(OH)D with adiposity remained statistically significant (*β* = –0.02, SE = 0.01, *p* = 0.04).

### 3.3. Analysis 3: Postnatal Body Size and Composition

In simple models, 25(OH)D at birth was not significantly associated total mass (*β* = 0.72, SE = 1.97, *p* = 0.71), fat-free mass (*β* = -0.27, SE = 1.24, *p* = 0.83), fat mass (*β* = 0.99, SE = 1.33, *p* = 0.46), or adiposity (*β* = 0.01, SE = 0.01, *p* = 0.41) at 5 months. Interactions of 25(OH)D with maternal BMI, GWG, breastmilk-months, and infant supplement use were also non-significant (data not shown). Use of infant supplements that contained vitamin D was not associated with body size or composition at 5 months (data not shown).

## 4. Discussion

In a diverse cohort of mother-offspring pairs, we found that cord blood 25(OH)D was associated with birth weight, but that the direction and magnitude of this association varied according to maternal pre-pregnancy BMI. Cord blood 25(OH)D was inversely associated with neonatal adiposity, although these findings were attenuated after adjustment for maternal BMI. There was no evidence of an association between cord blood 25(OH)D and infant body size or composition at 5 months of age. As expected, 25(OH)D at birth was predicted by both modifiable (maternal BMI, prenatal vitamin D intake) and non-modifiable (race/ethnicity, season of birth) factors.

Randomized clinical trials have shown that prenatal vitamin D supplementation increases both maternal 25(OH)D and offspring birthweight [8]. Given the decreased bioavailability of 25(OH)D observed in individuals with obesity due to sequestering in adipose tissue [20,28,29,30,31,32,33,34], we expected to find a positive association between 25(OH)D and birthweight that was attenuated with increasing BMI or GWG. In fact, we observed an inverse relationship: a trend toward lower birthweight with increasing 25(OH)D, but as BMI or GWG increased, this trend became increasingly positive in direction and magnitude. Among women with a higher BMI, increasing 25(OH)D was associated with increasing birthweight, while among women with a lower BMI, increasing 25(OH)D was associated with a decreasing birthweight. Prior observational studies have reported an increase in birthweight as cord blood 25(OH)D increases [56,57], a decrease in birthweight as cord blood 25(OH)D increases [19], and no association between cord blood 25(OH)D and birthweight [24,58]. Some [24,56,58], but not all [19,57], of these analyses were adjusted for maternal BMI, and none examined effect modification by maternal BMI. It is possible that the differential associations between 25(OH)D and birthweight according to maternal BMI we observed are due to variations in overall energy intake or other nutritional factors that are concomitant with increased vitamin D intake and thus increased 25(OH)D levels. Our finding that the association between cord blood 25(OH)D and birthweight varies according to maternal BMI must be replicated in other studies, including intervention trials, to determine if vitamin D supplementation recommendations to modify birthweight should be BMI-specific.

In contrast to our results for total mass at birth, we found that the association between 25(OH)D and neonatal adiposity did not differ by maternal pre-pregnancy BMI, but rather, was confounded by it. In a model not adjusted for maternal BMI, we observed a significant, inverse association between greater cord blood 25(OH)D and lower neonatal adiposity. After adjusting for maternal BMI, this association was attenuated to non-significance, which is in agreement with prior studies [22,24], and suggests that increasing maternal BMI accounts for both a decrease in circulating 25(OH)D that is available for placental transport [20,32] and increased neonatal adiposity [51]. These data underscore the increased risk for both early life vitamin D deficiency and increased adiposity conferred to offspring by women with obesity. Further research is needed to understand the long-term implications of these characteristics on offspring health.

The associations we observed between cord blood 25(OH)D and offspring body size and composition at birth did not persist to 5 months of age. This is consistent with a study of 108 Dutch infants in whom cord blood 25(OH)D was not related to change in infant weight from birth to 4 or 9 months of age [58], as well as a study of Asian offspring in whom maternal 25(OH)D status at 26–28 weeks gestation was not related to offspring size at 3–24 months [59]. However, there is limited evidence that early life 25(OH)D may be associated with offspring body size and composition later in childhood. The Southampton Women’s Survey reported that lower maternal 25(OH)D at 34 weeks gestation was independently associated with lower offspring fat mass at birth and higher fat mass at 6 years, with no independent association observed at 4 years [21]. Two other studies evaluating maternal 25(OH)D with offspring size and adiposity at 9 years reported null results [25,26]. Thus, the evidence surrounding the implications of low 25(OH)D at birth for future offspring adiposity is mixed, with variations in methodology, cohort characteristics, and analytic approaches compounding study differences. Future studies, including continued follow-up of the Healthy Start cohort, may help clarify the significance of early life 25(OH)D as children grow.

We also report the independent predictors of cord blood 25(OH)D, many of which are consistent with prior studies [20,22,24,56,57,60]. We observed the lowest 25(OH)D levels in offspring born of non-Hispanic black mothers, of mothers with higher pre-pregnancy BMI, or in the winter, while offspring born to non-Hispanic white mothers, to women with lower pre-pregnancy BMI, or in the summer had the highest 25(OH)D levels. These data are in agreement with results of prenatal vitamin D supplementation trials, which reported that even with supplementation, women with non-white race/ethnicity, higher pre-pregnancy BMIs, and winter deliveries had lower 25(OH)D levels in pregnancy [15,61]. For every 100 IU/day increase in vitamin D from food sources or dietary supplements, we observed a 0.6 nmol/L increase in cord blood 25(OH)D. This is a substantially smaller increase than the 2.5 nmol/L increase per 100 IU/day benchmark that has been used to guide vitamin D supplementation [62]. Based on our data, a non-Hispanic black mother expecting a winter baby would have to consume ~4400 IU/day more than a non-Hispanic white woman expecting a summer baby in order to have the same 25(OH)D levels in cord blood. This estimated dose increases even more with pre-pregnancy obesity. However, this dose also exceeds the United States National Academy of Sciences Food and Nutrition Board tolerable upper limit of 4000 IU/day of vitamin D for pregnant women [63], and therefore cannot be recommended based solely on the present results. It is possible that variations in absorption and specific sources of vitamin D (D_2_ versus D_3_) account for our differing estimate, and thus should be interpreted with caution. We also note that we did not measure maternal 25(OH)D during pregnancy, which has been shown to be significantly associated with cord blood 25(OH)D [20,22], and could have affected our estimates if that data had been available. Continued research into the implications of cord blood 25(OH)D status is needed to guide recommendations for prenatal vitamin D intake.

Our study has some limitations and several strengths. While Healthy Start is a large, diverse cohort, only a portion of the participants had sufficient data to be included in this present analysis, which may have reduced statistical power. Given that the analytic cohort was very similar to the full cohort in terms of maternal/offspring demographics, we do not believe that our analytic sample was biased in any way that would have affected the results. Because of the limited number of participants with complete data for all three analyses (*n* < 150), we were unable to conduct a longitudinal analysis, which should be addressed in future research. We did not have data on 25(OH)D during pregnancy or beyond birth and thus could not examine how changes in 25(OH)D over time may be related to offspring size and adiposity. We were only able to use crude estimates of offspring intake of vitamin D postnatally from formula or infant supplements, which may have prevented us from detecting associations between 25(OH)D at birth and 5-month body composition that varied by postnatal vitamin D intake. We used season of birth as a crude measurement of sun exposure because we did not have direct measures of UVB-synthesized vitamin D, and thus our results for the relative contribution of oral intake versus sun exposure should be interpreted with caution. The ethnically-diverse cohort, validated measures of 25(OH)D and body composition, and detailed data on maternal vitamin D intake during pregnancy are all strengths of our study.

In conclusion, we found that cord blood 25(OH)D levels are related to both modifiable and non-modifiable perinatal factors. Associations between 25(OH)D and offspring body size and composition at birth are modified by maternal BMI, but do not persist to 5 months of age. Future research should examine the implications of cord blood 25(OH)D for offspring growth and development during childhood and beyond.

## Figures and Tables

**Table 1:** Sample characteristics *.

	Mean (SD) or *N* (%)	
Maternal characteristics		
Age (years)	27.6	(6.3)
Race		
Hispanic	156	(26%)
Non-Hispanic white	328	(54%)
Black	87	(14%)
Other	34	(6%)
Education		
<High school degree	94	(16%)
High school degree	114	(19%)
A college or 2-year degree	139	(23%)
4-year degree	126	(21%)
Graduate degree	132	(22%)
Household income		
<$40,000	185	(31%)
$40,000–$70,000	112	(19%)
>$70,000	192	(32%)
Missing/do not know	116	(19%)
Pre-pregnancy BMI (kg/m^2^)	26.1	(6.5)
Gestational weight gain (kg)	14.0	(6.6)
Prenatal oral vitamin D intake (IU/day) (*n* = 305)	415	(453)
Neonatal characteristics		
Female (*n*)	289	(48%)
Gestational age at birth (weeks)	39.5	(1.1)
Season of birth		
Summer (June, July, August)	189	(31%)
Fall (September, October, November)	140	(23%)
Winter (December, January, February)	128	(21%)
Spring (March, April, May)	148	(24%)
Total 25(OH)D in cord blood (nmol/L)	56.0	(21.2)
Body size and composition		
Total mass (g)	3158	(412)
Fat-free mass (g)	2858	(332)
Fat mass (g)	300	(147)
Adiposity (%)	9.3	(3.8)
Postnatal characteristics (*n* = 348)		
Age at postnatal visit (months)	5.1	(1.3)
Exclusive breastfeeding (%)	151	(43%)
Use of infant vitamin D-containing supplements (%)	156.0	(30%)
Body size and composition		
Total mass (g)	6860	(881)
Fat-free mass (g)	5185	(626)
Fat mass (g)	1675	(507)
Adiposity (%)	24.1	(5.5)

SD: standard deviation. BMI: body mass index. * *n* = 605 unless otherwise noted.

**Table 2:** Predictors of total 25(OH)D (nmol/L) in cord blood (*n* = 305) *.

	Beta	(SE)	*p*-Value
Maternal pre-pregnancy BMI (per 5 kg/m^2^)	–3.2	(0.8)	<0.001
Race/ethnicity			
Non-Hispanic white	1.0	(ref)	
Non-Hispanic black	–15.6	(3.4)	<0.001
Hispanic	–8.2	(3.0)	0.006
Daily prenatal oral vitamin D intake (per 100 IU)	0.6	(0.2)	0.01
Season of birth			
Summer	1.0	(ref)	
Fall	–6.8	(2.9)	0.02
Winter	–12.1	(3.1)	<0.001
Spring	–10.4	(2.9)	<0.001

* Adjusted for maternal age at delivery, household income, gestational weight gain, and gestational age at birth.

**Table 3:** Associations between cord blood 25(OH)D and offspring body size and composition at birth (*n* = 605) *.

	Simple Model			Simple Model + BMI			Simple Model + GWG		
	Beta	(SE)	*p*-Value	Beta	(SE)	*p*-Value	Beta	(SE)	*p*-Value
Total mass (g)									
Total 25(OH)D (nmol/L)	–1.44	(0.76)	0.06	–6.22	(2.67)	0.02	–5.13	(1.68)	0.002
Pre-pregnancy BMI (5 kg/m^2^)				2.08	(27.11)	0.94			
Total 25(OH)D × BMI				1.05	(0.50)	0.04			
Gestational weight gain (kg)							–4.19	(6.13)	0.49
Total 25(OH)D × GWG							0.26	(0.11)	0.02
Fat-free mass (g)									
Total 25(OH)D (nmol/L)	–0.91	(0.59)	0.12	–0.58	(0.60)	0.33	–3.74	(1.31)	0.005
Pre-pregnancy BMI (5 kg/m^2^)				28.64	(9.46)	0.003			
Gestational weight gain (kg)							–3.86	(4.79)	0.42
Total 25(OH)D x GWG							0.20	(0.09)	0.02
Fat mass (g)									
Total 25(OH)D (nmol/L)	–0.48	(0.30)	0.11	–0.19	(0.30)	0.51	–0.52	(0.30)	0.08
Pre-pregnancy BMI (5 kg/m^2^)				24.75	(4.71)	0.0001			
Gestational weight gain (kg)							2.72	(0.90)	0.003
Adiposity (%)									
Total 25(OH)D (nmol/L)	–0.02	(0.01)	0.05	–0.01	(0.01)	0.25	–0.02	(0.01)	0.04
Pre-pregnancy BMI (5 kg/m^2^)				0.57	(0.12)	0.0001			
Gestational weight gain (kg)							0.05	(0.02)	0.02

* Adjusted for maternal age at delivery, race/ethnicity, household income, offspring sex and gestational age at birth.
